# Real-Time Vital Mineralization Detection and Quantification during In Vitro Osteoblast Differentiation

**DOI:** 10.1186/s12575-018-0079-4

**Published:** 2018-08-01

**Authors:** Anastassia Serguienko, Meng Yu Wang, Ola Myklebost

**Affiliations:** 1 Institute for Cancer Research, Oslo University Hospital, Po Box 4950 Nydalen, 0424 Oslo, Norway; 2 Department of Clinical Science, University of Bergen, Po Box 7804, 5020, Bergen, Norway

**Keywords:** Osteoblast differentiation, Bone, Mesenchymal, Mineralization, Calcein green, Time-lapse photography, Imaging, Alizarin red

## Abstract

**Background:**

In vitro studies of osteoblasts traditionally use Alizarin Red as a golden standard for the detection and quantification of mineralization, which is a marker of osteoblast differentiation. However, this method presents a number of drawbacks, including the need to fix cells, which prevents additional measurements. Years ago, Calcein Green was proposed as an alternative to Alizarin Red, with the advantage to be directly detectable in live cells. However, the protocol was still time-consuming, and it never managed to replace Alizarin Red. Now, with more efficient imaging systems, we present a protocol using Calcein Green which provides significant advantages.

**Results:**

The osteoblast mineralization was efficiently detected and accurately quantified in real time at any desired time point across the entire differentiation period, with a minimum time expenditure.

**Conclusions:**

The combination of Calcein Green and the real-time imaging station IncuCyte ZOOM can efficiently replace the Alizarin Red method, and allows very accurate and time-saving assessment of the level and the dynamics of matrix mineralization.

**Electronic supplementary material:**

The online version of this article (10.1186/s12575-018-0079-4) contains supplementary material, which is available to authorized users.

## Background

The availability of in vitro model systems has led to detailed understanding of the biological processes that govern bone development. Primary mesenchymal stem cells (pMSCs) derived from various tissues can be cultured and induced to differentiate to osteoblasts in vitro, allowing studies of the molecular mechanisms involved. The final stage, matrix deposition and mineralization, is traditionally used as readout of the differentiation efficiency, but its quantification has been cumbersome and requires termination of the cultures. The standard method consists of the fixation of the cells with formalin, followed by staining with Alizarin Red dye, and inspection in the phase-contrast microscope. For quantitative analysis, the color can be extracted and quantified by a colorimetric assay.

In spite of several drawbacks, this method is still dominating in vitro mineralization research. Since the cells have to be fixed, only one time point per sample can be analyzed, the moderate sensitivity makes early differentiation hard to detect, and the reproducible removal of background signal from non-specific dye binding is difficult. The obtained absorbance has to be normalized to the cell number, most commonly by measurement of total protein in a parallel sample, which increases variability, and the method requires several hours of lab work.

Calcein Green (hereafter calcein) is a cell-non-permeant dye that fluoresces when bound to calcium crystals. It has been proposed for the detection of calcium hydroxyapatite 15 years ago [1]. However, although it is used for in vivo experiments, it is not applied in differentiation research on cultured osteoblasts [2]. The advances in the development of laboratory equipment and technology now makes this method much more useful.

We have adapted calcein quantification to the real-time, automated IncuCyte ZOOM live-cell imaging station, which automatically acquires, analyzes and quantifies images. The machine is provided with a phase contrast microscope with red and green fluorescence modes. Combining this imaging station with the use of calcein turns out to be a superior method for studying osteoblast differentiation in vitro. This method eliminates the above mentioned disadvantages of the Alizarin Red, and allows to collect in real-time the data, otherwise not achievable.

## Methods

### Cell Culture

Primary MSCs were isolated from bone marrow of a female donor [3]. Cells were cultured in α-MEM medium containing Glutamax (catalogue N 32561–029, Gibco), supplemented with 15% fetal bovine serum. Cells were kept in culture for no more than 3 passages, and the medium was refreshed every four days.

### Induction of Osteoblast Differentiation

Osteogenic cocktail was prepared as followed: 10 nM dexamethasone, 3.5 mM beta-glycerol phosphate, 50 μg/ml ascorbic acid (all purchased from Sigma-Aldrich). Cocktail- or vehicle-treated cells were seeded on day 0 and never split until the termination of the experiment. The cocktail- or vehicle-containing medium was refreshed every four days.

### Transient Transfection

pMSC were seeded in a 12-well plate at 30% of confluence per well one day before the transfection. The transfection mix was prepared using 18 nM of PPP2R2C siRNA (catalogue N AM17111, Ambion) or negative control siRNA (catalogue N AM16704, Ambion) and 7.5 μl of lipidic transfection reagent Interferin (catalogue N 409–50, Polyplus) (quantities per well). The transfection mix was added to the cells overnight and removed the next morning by completely replacing the medium. For long-term experiments, the transfection was repeated every four days.

### Alizarin Red Staining and Calcium Deposit Quantification

Cells were fixed with 10% formalin solution for 20 min at room temperature (RT) and rinsed with PBS. 40 mM solution of Alizarin Red (catalogue N 5533-25G, Sigma-Aldrich) was added to the cells for 30 min at RT with agitation. Next, unbound Alizarin Red dye was removed, and the cells were washed several times with Milli-q water. Alizarin Red staining was detected by the Olympus IX2-UCB microscope in the phase-contrast setting.

For calcium quantification, 10% acetic acid (*v*/v) was added to the cells stained with Alizarin Red and incubated for 30 min at RT with agitation. Cells were scraped off, transferred to Eppendorf tubes, vortexed for 30 s, and incubated for 10 min at 85 °C. Next, samples were centrifuged for 15 min at 16 g and 200 μl of the supernatant was transferred to another Eppendorf tube. 22.5 μl of 10% NH_4_OH (*v*/v) was added to the samples and mixed. The absorbance was measured at 405 nm. Values were normalized to a calibration curve.

### Calcein Green Staining

From powder calcein (catalogue N *C0875*, Sigma-Aldrich) 10 mM calcein solution was prepared in 0.1 M of NaOH; it was further diluted 1:10 in ddH_2_O in order to have a 1 mM working stock. Final solution, 1 μM (1:1000), can be increased up to 2 μM (1:500 dilution). The working solution was sterile filtered, using 0.2 μm pore filter. Calcein solution was added directly to the differentiation medium first on day 4 of differentiation, and subsequently, each time the medium was refreshed. To further reduce the fluorescent background, medium containing calcein can be replaced with calcein free medium before taking images. Green fluorescence was detected with an Olympus IX2-UCB fluorescent microscope for 500 ms, ISO 200, laser intensity 25.

### IncuCyte ZOOM

The green fluorescence mask was set in order to pick out only very small green dots excluding aggregates to avoid artefacts. An example mask: Threshold adjustment GCU: 35 Edge sensitivity: − 44, Area, μm: min 35 max 122.09, Mean intensity: min 70.3. However, the mask needs to be adjusted for every single independent experiment.

## Results

**Fig. 1:**
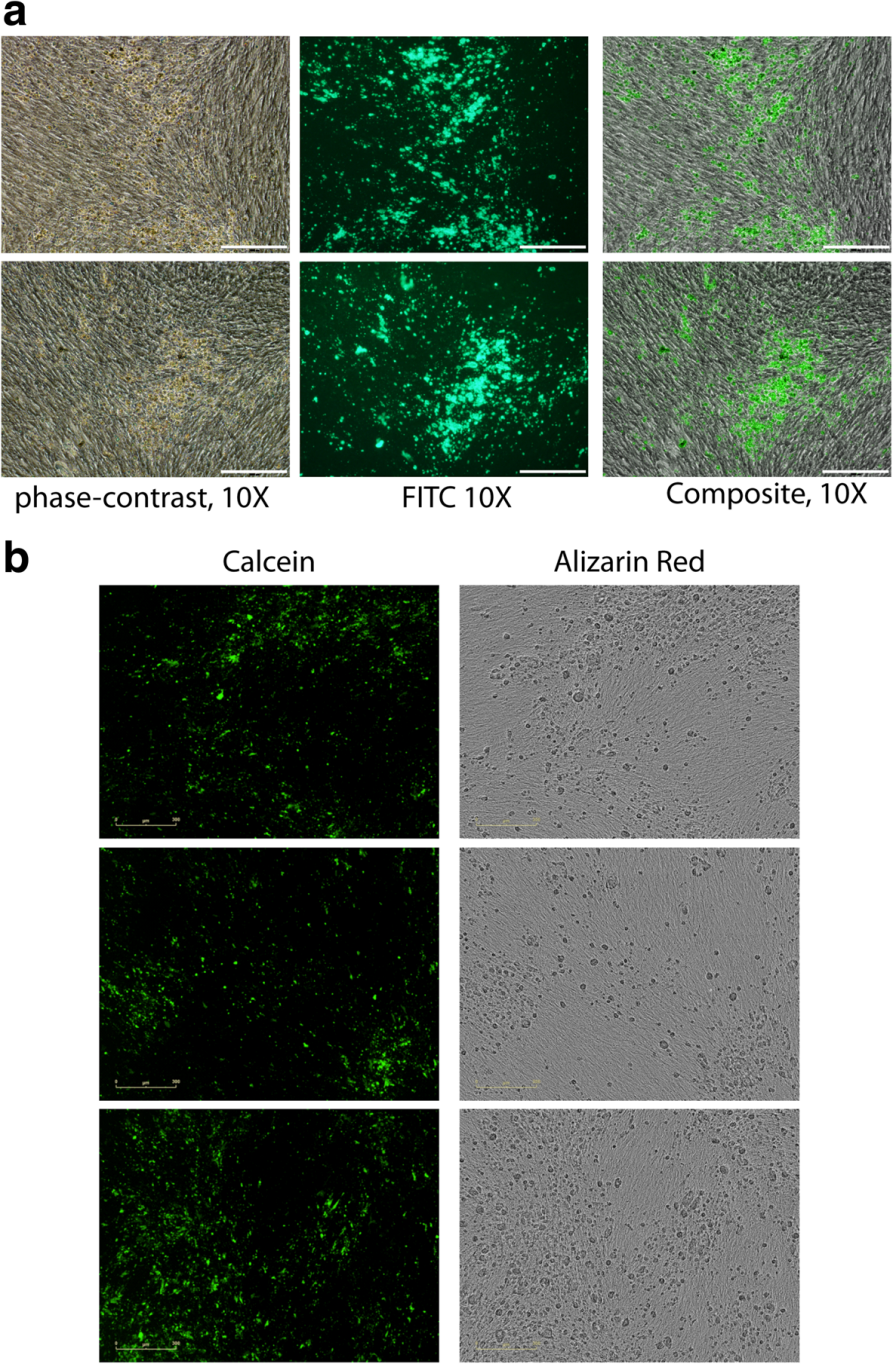
Comparison of the efficiency of calcein versus Alizarin Red to detect mineralized areas of osteoblast culture. **a** mineralization pattern detected by Olympus IX2-UCB Fluorescent microscope, phase-contrast (left panel, calcium hydroxyapatite deposits appear like yellowish structures at phase-contrast); the same section on the plate visualized by calcein by fluorescent setting (middle panel); overlap between the phase-contrast and fluorescent images in composite setting (right panel), scale bars 2000 μm. Representative pictures are shown, *n* = 3. **b** Calcein staining and Alizarin Red staining of the same mineralized areas detected by IncuCyte ZOOM: note that the phase-contrast objective of the IncuCyte ZOOM does not show the red color of Alizarin Red, however the pattern of the staining is clearly visible in gray scale and can be compared to the calceinpattern; representative pictures are shown, *n* = 3

**Fig. 2:**
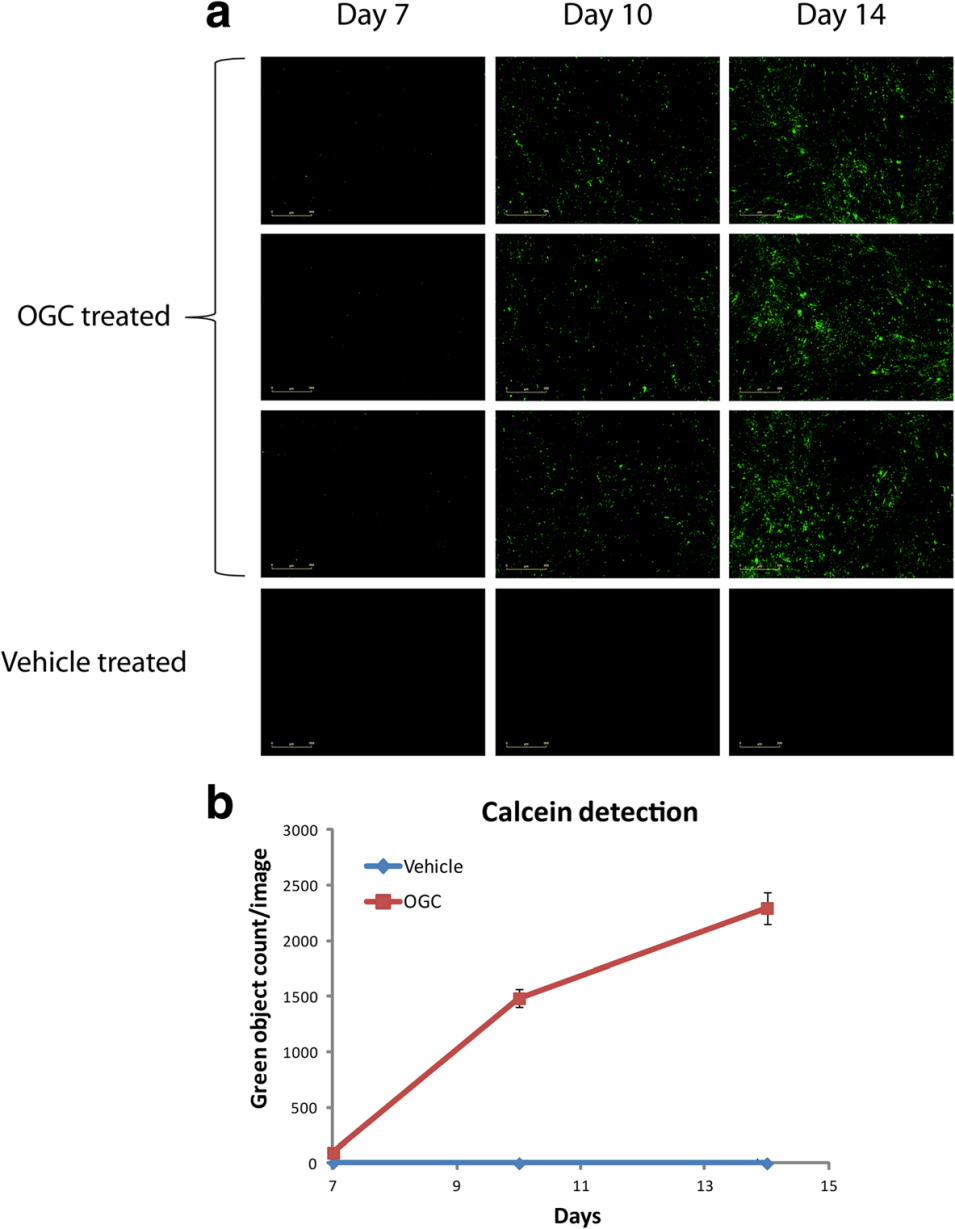
Mineralization dynamics of differentiating osteoblasts treated with osteogenic cocktail (OGC), detected by Calcein. **a** Fluorescent images of the cell culture obtained by IncuCyte ZOOM at day 7, day 10 and day 14 of differentiation. Last row: vehicle (ethanol) treated cells, showing no differentiation, representative pictures are shown, *n* = 3. **b** Mineralization curve generated automatically by the IncuCyte ZOOM based on the green object count per image. The cells were scanned automatically every 3 h for two weeks, representative experiment is shown, *n* = 3, SE

**Fig. 3:**
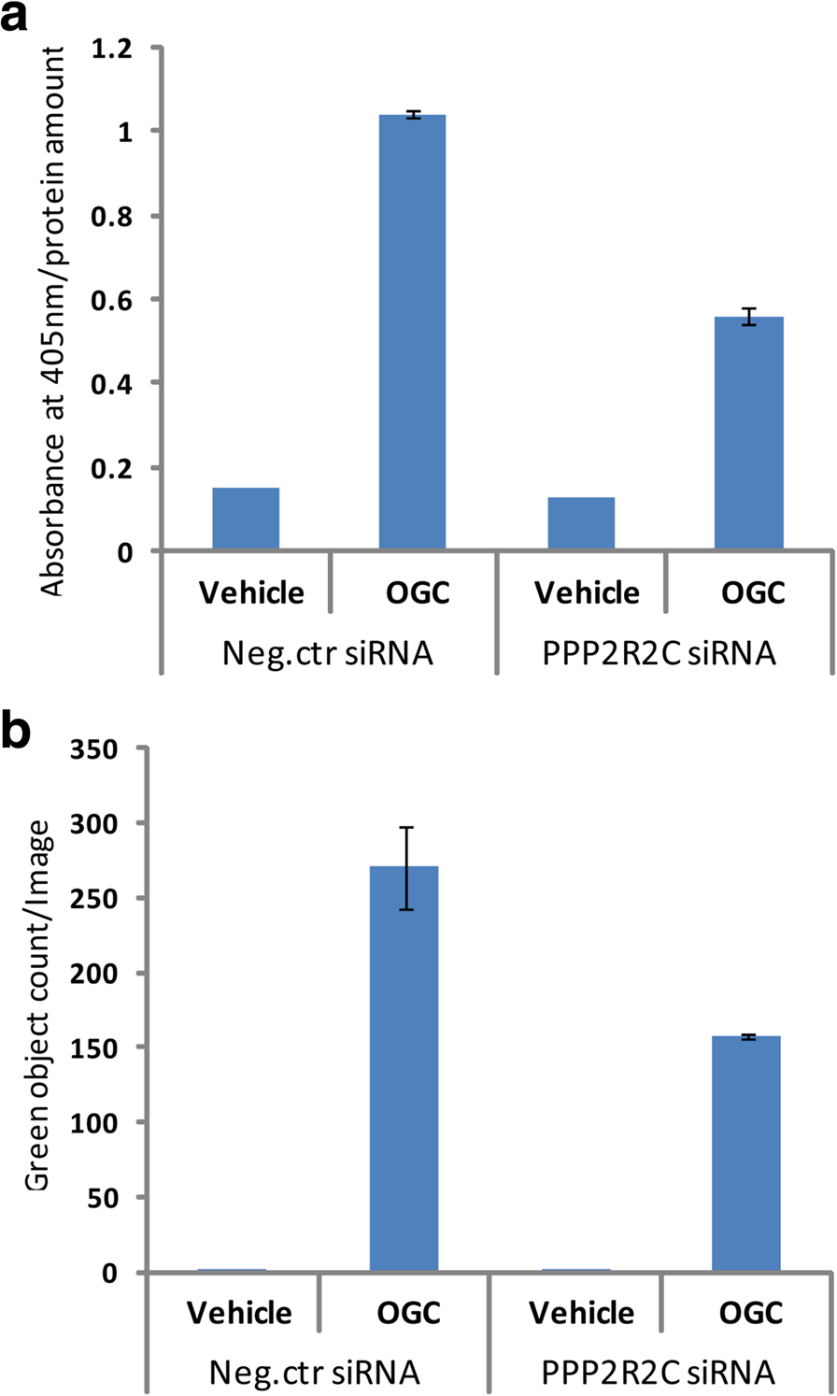
Comparison of quantification of mineralization obtained either by Alizarin Red (**a**) or calcein (**b**). MSCs were transfected by an siRNA against PPP2R2C and the mineralization was quantified on day 12 of osteoblast differentiation **a** Measurement of extracted Alizarin Red, **b** Measurement of calceinin cultures in the IncuCyte ZOOM. MSCs transfected with negative control siRNA were used as a control, *n* = 2, SE

Primary mesenchymal stem cells induced to differentiate to osteoblasts were incubated at 37 degrees and monitored in the presence of Calcein Green for 14 days in the IncuCyte ZOOM. The phase-contrast and fluorescent pictures were automatically obtained across the indicated periods. The pattern of calcium hydroxyapatite deposition, detected by the Calcein Green staining, overlapped completely with those observed by phase-contrast (Fig. 1a). At the end of the differentiation period (day 14) the cells previously treated with Calcein Green were fixed and further stained with Alizarin Red. The images were obtained by the IncuCyte ZOOM and compared with the calcein images previously obtained from the same cells. Although the IncuCyte ZOOM does not visualize the red colour, the pattern of Alizarin Red staining is clearly visible and can be compared to that of calcein Green (Fig. 1b). Next, we assessed the dynamics of the differentiation. Differentiating osteoblasts were followed in the IncuCyte ZOOM for 14 days. The differentiation medium was refreshed every 4 days. The deposition of calcium hydroxyapatite crystals was detected by fluorescence at days 7, 10, and 14. Images were automatically saved, and the line chart with the quantification of mineralization over the observed period was automatically generated after the application of the properly set mask (Fig. 2a, b and Additional file 1: Figure S1a). Importantly, Calcein Green does not generate any permanent background or false positive images, as determined from vehicle-only treated samples and additionally by the comparison of differentiation cocktail treated cells with and without calcein (Additional file 1: Figure S1b). To remove completely background fluorescence, the medium can be replaced with calcein-free medium immediately before scanning. Next, we assessed whether the quantification of calcium deposits by the Alizarin Red color extraction, can be reliably replaced by the automatic quantification of calcein performed in the IncuCyte ZOOM. We transfected pMSCs with the negative control siRNA or with siRNA against PPP2R2C, that we previously showed to be required for osteoblast differentiation [4]. Transfected cells were treated with the osteogenic cocktail or vehicle on day 1 after transfection. Subsequently, the cells were re-transfected every 3 days. On day 12, half of the wells were stained with Alizarin Red and the color was extracted and quantified (Fig. 3a). The staining of the other half with calcein was measured directly from cultures by the Incucyte Zoom, by the counting of green objects per image (Fig. 3b and Additional file 1: Figure S1c). The quantification by Alizarin Red was similar to the quantification in the IncuCyte ZOOM, except that the background level in control samples was much lower with calcein objects count (0.02 calcein versus 0.15 Alizarin Red). Note, that by the background level in the calcein samples we mean the number of false positive objects, while in the Alizarin Red samples, it is absorbance values.

Long term or periodic incubations with calcein does not affect cell viability, which was assessed by the dead cells count using propidium iodide (Additional file 1: Figure S1c).

## Discussion

Thus, the Alizarin Red method can be fully replaced by the combined use of calcein and IncuCyte ZOOM for in vitro studies of osteoblast differentiation. The major advantage brought by this technique is the possibility to monitor deposition rates in real-time in living cells, a significant time saving, avoiding the need to compare wells when additional measurements are done, as well as a very low background, both latter aspects improving accuracy. Importantly, calcein is cell-non-permeant, therefore it does not interfere with normal cell physiology and does not produce any intracellular fluorescence.

## Conclusions

Monitoring bone mineralization during in vitro studies of osteoblast differentiation by calcein fluorescence detection with automatic time-lapse equipment is superior to traditional Alizarin Red staining, and much more convenient.

## Additional file

Additional file 1: **Figure S1.** **a** An example of the mask set for the green objects count on day 10; left pictures shows green fluorescent dots corresponding to the forming calcium crystals; right pictures shows how the IncuZOOM mask depicted green dots with high sensitivity (even very weak and small green dots are counted), representative pictures are shown. **b** Real-time calcium crystals formation detected by fluorescent imaging in calcein-treated and calcein-untreated samples, *n* = 3, SE. **c** Calcein detection in negative control and PPP2R2C siRNA treated samples, representative pictures are shown. **d** Number of dead cells detected by propidium iodide in calcein-treated and calcein untreated cells, *n* = 2, SE. (TIF 2900 kb)
