# Supplementary figures and images for: Real-Time Vital Mineralization Detection and Quantification during In Vitro Osteoblast Differentiation
Source: Biol Proced Online. 2018 Aug 1;20:14. doi: 10.1186/s12575-018-0079-4 (PMC6069543; doi:10.1186/s12575-018-0079-4)

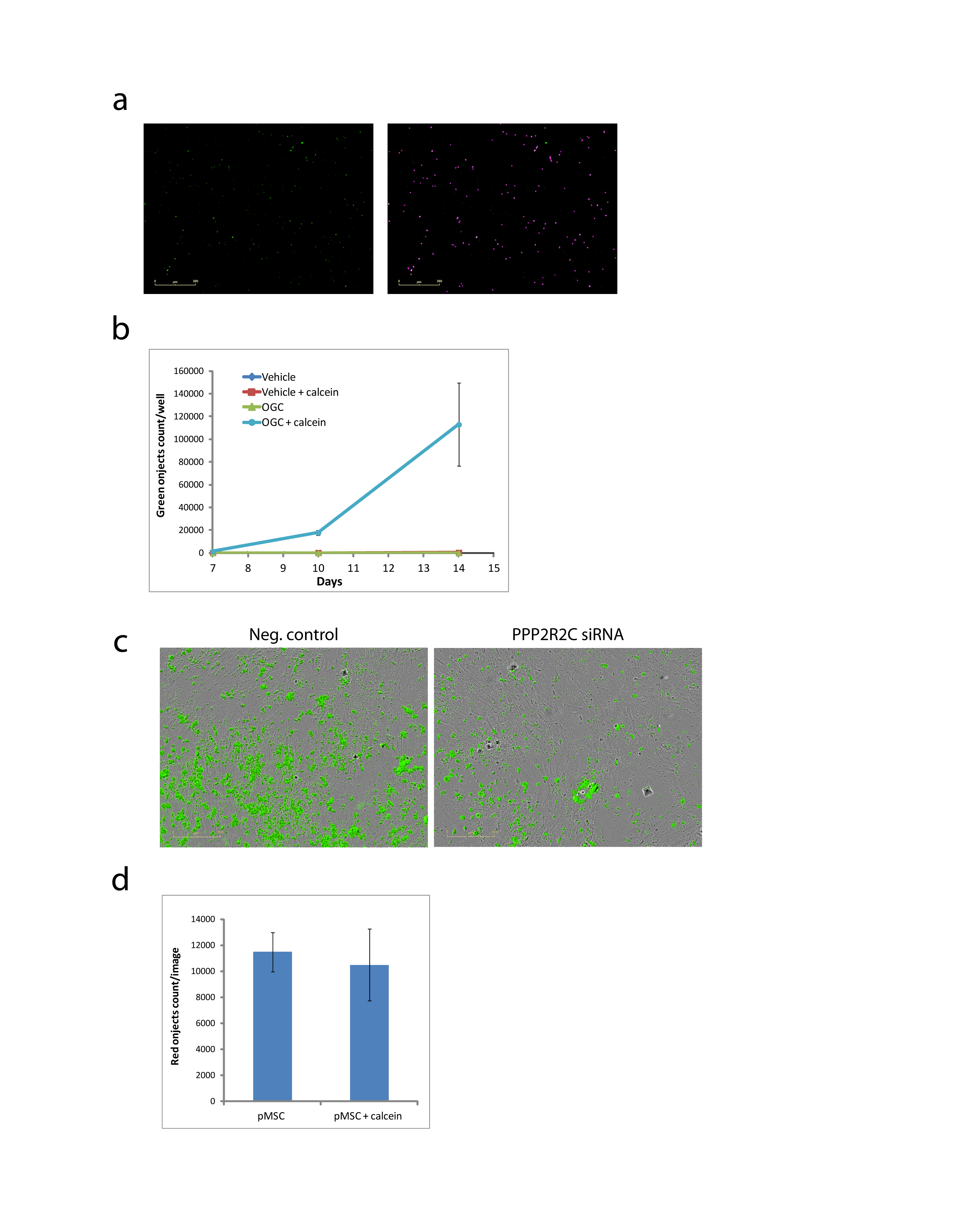

Supplement: Supplementary file 1 — Figure S1. a An example of the mask set for the green objects count on day 10; left pictures shows green fluorescent dots corresponding to the forming calcium crystals; right pictures shows how the IncuZOOM mask depicted green dots with high sensitivity (even very weak and small green dots are counted), representative pictures are shown. b Real-time calcium crystals formation detected by fluorescent imaging in calcein-treated and calcein-untreated samples, n = 3, SE. c Calcein detection in negative control and PPP2R2C siRNA treated samples, representative pictures are shown. d Number of dead cells detected by propidium iodide in calcein-treated and calcein untreated cells, n = 2, SE. (TIF 2900 kb) [file 12575_2018_79_MOESM1_ESM.tif]
